# In-Vitro Evaluation of Photofunctionalized Implant Surfaces in a High-Glucose Microenvironment Simulating Diabetics

**DOI:** 10.3390/jfb14030130

**Published:** 2023-02-26

**Authors:** Supriya Kheur, Mohit Kheur, Vaibhav Madiwal, Ramandeep Sandhu, Tabrez Lakha, Jyutika Rajwade, Tan Fırat Eyüboğlu, Mutlu Özcan

**Affiliations:** 1 Department of Oral Pathology and Microbiology, Dr. D. Y. Patil Dental College and Hospital, Dr. D. Y. Patil Vidyapeeth, Pune 411044, India; 2 Department of Prosthodontics, M.A. Rangoonwala College of Dental Sciences and Research Centre, Pune 411001, India; 3 Nanobioscience Group, Agharkar Research Institute, Pune 411004, India; 4 Department of Fixed Prosthodontics, Holland Bloorview Kids Rehabilitation Hospital, University of Toronto, Toronto, ON M4G 1R8, Canada; 5 Department of Endodontics, Faculty of Dentistry, Istanbul Medipol University, 34083 Istanbul, Turkey; 6 Head Division of Dental Biomaterials, Director a.i. Clinic of Masticatory Disorders, Center of Dental Medicine, Clinic of Reconstructive Dentistry, University of Zurich, CH-8032 Zurich, Switzerland

**Keywords:** dental implants, dental materials, diabetes mellitus, osteoblasts, photofunctionalization, prosthodontics, titanium

## Abstract

The present study aimed to assess the efficacy of photofunctionalization on commercially available dental implant surfaces in a high-glucose environment. Discs of three commercially available implant surfaces were selected with various nano- and microstructural alterations (Group 1—laser-etched implant surface, Group 2—titanium–zirconium alloy surface, Group 3—air-abraded, large grit, acid-etched surface). They were subjected to photo-functionalization through UV irradiation for 60 and 90 min. X-ray photoelectron spectroscopy (XPS) was used to analyze the implant surface chemical composition before and after photo-functionalization. The growth and bioactivity of MG63 osteoblasts in the presence of photofunctionalized discs was assessed in cell culture medium containing elevated glucose concentration. The normal osteoblast morphology and spreading behavior were assessed under fluorescence and phase-contrast microscope. MTT (3-(4,5 Dimethylthiazol-2-yl)-2,5-diphenyltetrazolium bromide) and alizarin red assay were performed to assess the osteoblastic cell viability and mineralization efficiency. Following photofunctionalization, all three implant groups exhibited a reduced carbon content, conversion of Ti4+ to Ti3+, increased osteoblastic adhesion, viability, and increased mineralization. The best osteoblastic adhesion in the medium with increased glucose was seen in Group 3. Photofunctionalization altered the implant surface chemistry by reducing the surface carbon content, probably rendering the surfaces more hydrophilic and conducive for osteoblastic adherence and subsequent mineralization in high-glucose environment.

## 1. Introduction

The success of implant integration and function depends upon good bone-to-implant contact (BIC). One of the most important factors for good osseointegration is the physical and chemical nature of the implant surface. The purpose of modifying the implant surfaces is to improve osseointegration. This process has been a subject of continued research focus. It enhances the biological surface properties of implants, which in turn, favors osseointegration. Implant surfaces have been modified using different mechanical and chemical strategies such as etching using acid, blasting through a grit and/or sandblasting, biomimetic coating, and anodizing [1,2,3,4]. Additional modalities include bioactive molecules, including growth factors (platelet-derived growth factor, bone morphogenetic protein, vascular endothelial growth factor), inorganic materials (HA, calcium phosphate), extracellular matrix components (collagen, chondroitin sulfate, hyaluronic acid), and peptides [1,2,3,4,5]. 

Additionally, treating the titanium implant surface with plasma or UV irradiation (photo-functionalization) before its use has also been investigated, where UV-A and UV-C wavelengths were reported to increase the hydrophilicity of the surface by removing the carbon from the titanium surface and converting Ti4+ to Ti3+, thereby inducing nanoscale surface modification [6,7]. Plasma treatment of implants improves initial osseointegration of implants by increasing the wettability and subsequently osteoblastic cell adhesion [7]. Research over the past decade has shown the ability of photo-functionalization to transform a hydrophobic implant surface into a super-hydrophilic surface capable of enhanced osteoconductivity, and data reporting photofunctionalization of dental implants ranges from in vitro, in vivo animal-based studies (rats, beagle dogs) to human clinical trials [8,9,10,11]. In rat models, titanium samples (machined and acid-etched) subjected to UV irradiation for 48 h presented a substantial increase in osteoblast proliferation, differentiation, spread, and attachment after UV treatment [2]. The histomorphometry showed the implants to exhibit significant bone formation, with a maximum bone–implant ratio achievement as early as in the 4th week of healing [2], which can increase from 45% to 95% [12].

Systemic conditions, including diabetes mellitus, osteoporosis following bisphosphonate treatment and radiotherapy, are known to retard the osseointegration of dental implants by affecting bone quality and quantity [13]. Diabetes mellitus is a major metabolic disorder with a well-established global prevalence that causes a state of hyperglycemia, marked by a decrease in bone formation markers, specifically bone-specific alkaline phosphatase (bALP) [14,15,16,17] and procollagen type 1N- terminal propeptide (PINP) and osteocalcin [17]. In vitro studies have shown that increased blood glucose levels (fasting plasma glucose > 126 mg/dL) alter osteoblast function, including proliferation, migration, and adherence [17,18]. Osseointegration and its maintenance require a coordinated function of osteoblasts and osteoclasts, which in turn is regulated by the action of osteocytes [18]. Increased glucose level also leads to altered osteocyte function. Animal experiments have shown significantly lower BIC in diabetes [19,20]. Clinically, the success rate of implants is not altered in type 2 diabetic patients, but stringent glucose control is the primary criteria for achieving predictable osseointegration [20]. Others have reported significantly greater marginal bone loss at implants placed in diabetic subjects [5,21,22].

The potential benefits of photofunctionalization of dental implants placed in diabetic patients are not well established. One study reported improved strength of osseointegration at photofunctionalized implants at weeks 2 and 4 in diabetic rats [23]. Photofunctionalization as a pre-placement procedure applied to commercially available implant surfaces may counter, to an extent, the impaired osseointegration process inherent to diabetes [18]. Thus, the present in vitro study was formulated to assess the effects of photofunctionalization of dental implant surfaces on the response of osteoblasts under conditions that simulate diabetes.

## 2. Material and Methods

Three commercially available titanium implant surface discs were provided by the respective manufacturer. Three samples of each group (n = 3 for each group) were randomly assigned to one of these groups to eliminate bias. These were provided in the sterile packages by the manufacturer in the dimension of diameter 10 mm and thickness of 1 mm. The packages were only opened at the start of the experiments. The study groups were as follows:

Group 1—laser-etched implant surface (Laser Lok, BioHorizons, Birmingham, AL, USA);

Group 2—titanium–zirconium alloy surface (Roxolid, Straumann Implants AG, Basel, Switzerland);

Group 3—air-abraded, acid-etched surface (SLA dental implants, Straumann Implants AG, Basel, Switzerland).

Untreated implant surfaces for each group were taken as control surfaces for that group. All the experiments were performed in triplicate to eliminate bias.

### 2.1. Photofunctionalization Unit

The specimens in all the groups were treated using a customized photofunctionalization unit (Lelesil Innovative systems, Thane, India) for 60 and 90 min. The light source used was a 125-watt and 250-watt medium-pressure mercury (Hg) lamp with dual lamp monitoring. The wavelength of the light used for UV-A was 345–400 nm and UV-C was 250–260 nm [24]. The whole assembly was mounted on a UV-protected safety cabinet with a water-cooling facility.

### 2.2. X-ray Photoelectron Spectroscopy (XPS) Based Implant Surface Analysis

All three groups of implants were subjected to XPS before and after photofunctionalization to assess for changes in the surface chemical composition. The spot, analyzed at 7.5 mm, was the center of the implants, and the spot size was 300 μm. The analysis was carried out using a custom-built ambient-pressure XPS system from Prevac equipped with a Scienta monochromator (MX650) using an Al Kα anode (1486.6 eV). The energy of the photoelectrons was determined using a Scienta R3000HP differentially pumped analyzer [25].

### 2.3. Cell Viability and Attachment Assay

The viability of MG63 (osteoblasts) cells was assessed by 3-(4, 5-dimethylthiazol-2-yl)-2-5 diphenyl tetrazolium bromide (MTT) (Sigma, Bangalore, India) assay. The MG63 cell line was procured from the National Centre for Cell Science (NCCS), Pune, India. During assay, 2 × 10^4^ cells per implant were seeded in a 24-well plate. The cells were incubated at 37 °C in a humidified atmosphere containing 5% CO_2_ for 48 h. After 48 h, the media were removed, followed by the addition of freshly prepared MTT reagent (stock concentration 5 mg/mL), and incubation was continued for 4 h. Subsequently, reagent containing medium was removed, and 0.5 mL DMSO (Sisco Research Laboratories, Pvt. Ltd., Mumbai, India) was added to dissolve the insoluble formazan crystals. A multi-well plate reader (Synergy HT, Bio-Tek Instruments Inc., Winooski, Vermont, USA) was employed to measure the absorbance at 570 nm. The formula used to calculate the cell viability percentage is as follows:

(1) $$\%Cell\;viability=\frac{OD570nm\;of\;treated\;cells\times 100}{OD570nm\;of\;control}$$

Cell morphology was assessed using an inverted phase-contrast microscope (Carl Zeiss Inc., Gottingen, Germany). Spreading behavior and cytoskeleton arrangement of osteoblasts seeded onto photofunctionalized titanium surfaces were examined using confocal laser scanning microscopy. After 24 h of seeding, cells were fixed in 4% paraformaldehyde and stained using fluorescent dye rhodamine–phalloidin (actin filament, red color; Molecular Probes, Eugene, OR, USA). The MG-63 osteoblasts were inoculated in DMEM medium containing high-glucose concentration ~25 mM, indicating diabetic conditions. Microscopic observations and cell viability were determined as described earlier.

### 2.4. Mineralization Assay

Alizarin red S (ARS) staining is a well-documented method to assess the deposition of calcium. For this assay, untreated (control) and photo-functionalized implant surfaces were seeded with 2 × 10^4^ cells in 24-well plates containing DMEM (10% FBS and 1% antibiotic supplementation) and incubated for 21 days. Cells were incubated in a humidified atmosphere with replenishment of culture media periodically. Cultures on day 21 were washed with PBS. The monolayer was then fixed using 4% (*w*/*v*) paraformaldehyde (Sigma–Aldrich) for 15 min at 37 °C. Distilled water was used to wash the monolayers twice. This was followed by addition of 40 mM ARS (1 mL) (pH 4.1) to each of the wells. By shaking gently, incubation of the plates was carried out for 20 min at 37 °C. After incubation, the excess dye was removed. Distilled water was employed to wash the well 5 times. Before extraction of the dye, the samples were stored at −20 °C. ARS was extracted from the monolayer by incubating in 0.5 mL 10% cetyl pyridinium chloride (CPC) solution for one hour. Then, 200 µL aliquots was transferred to a 96-well plate prior, and absorbance was recorded at 405 nm.

### 2.5. Statistical Analysis

Data obtained were compiled in an MS Office Excel Sheet (v 2010, Microsoft Redmond Campus, Redmond, WA, USA). Statistical package for social sciences (SPSS v21.0, IBM, Bangaluru, India) was used for the data analysis. Descriptive statistics were determined, and non-parametric tests were used for comparisons. Mann–Whitney U test was employed for 2-group comparison. Kruskal–Wallis was used for multiple-group comparison. In XPS analysis, as the data were descriptive, no statistical analysis was performed. Means of the values were taken for comparative analysis in the group at different time frames. *p* < 0.05 was considered statistically significant in all tests.

## 3. Results

### 3.1. XPS Implant Surface Analysis

The XPS survey revealed the presence of various elements such as carbon (C), oxygen (O), and titanium (Ti) as the major components, whereas aluminum (Al,) calcium (Ca) and chloride (Cl) were the minor components, with traces of Si and N. There was a dominance of Ti and O signals, which are indicative of the presence of a titanium oxide layer on the surface. The strong C signal on the untreated surface showed contamination due to adsorbed carbon containing organic molecules. The C1s was found to vary somewhat in both intensity and shape from sample to sample. The C1s peak is always dominated by the peak at a binding energy of 285 eV, which corresponds to hydrocarbon (C-H and C-C) bonded carbon.

XPS analysis revealed a significant reduction of the C (carbon) specific peak after UV treatment of the surfaces. Post UV photofunctionalization, a reduction in the C1s peak in Group 1 was observed (48% and 63% reduction after exposure for 60 and 90 min, respectively). The UV photofunctionalization showed a reduction in carbon content on the surface of all three groups, with results more prominent at the 90 min time interval (Table 1).

Broad O1s core level spectra consisting of two distinct features with a low intensity hump at high BE energy were observed in all the groups. The spectra were formally deconvoluted. The peak at 530.1 eV can be associated with metal oxide, such as TiO or CaO. The metal oxide feature was observed as the major peak component for Groups 2 and 3 implant surfaces. The groups at other time frames showed a reduction from the baseline samples as received (Table 1). The XPS spectra showed an increase in the peaks of O 1 s after 60 and 90 min of UV photofunctionalization in all groups, but significant results were observed only after 90 min.

**Figure 1:**
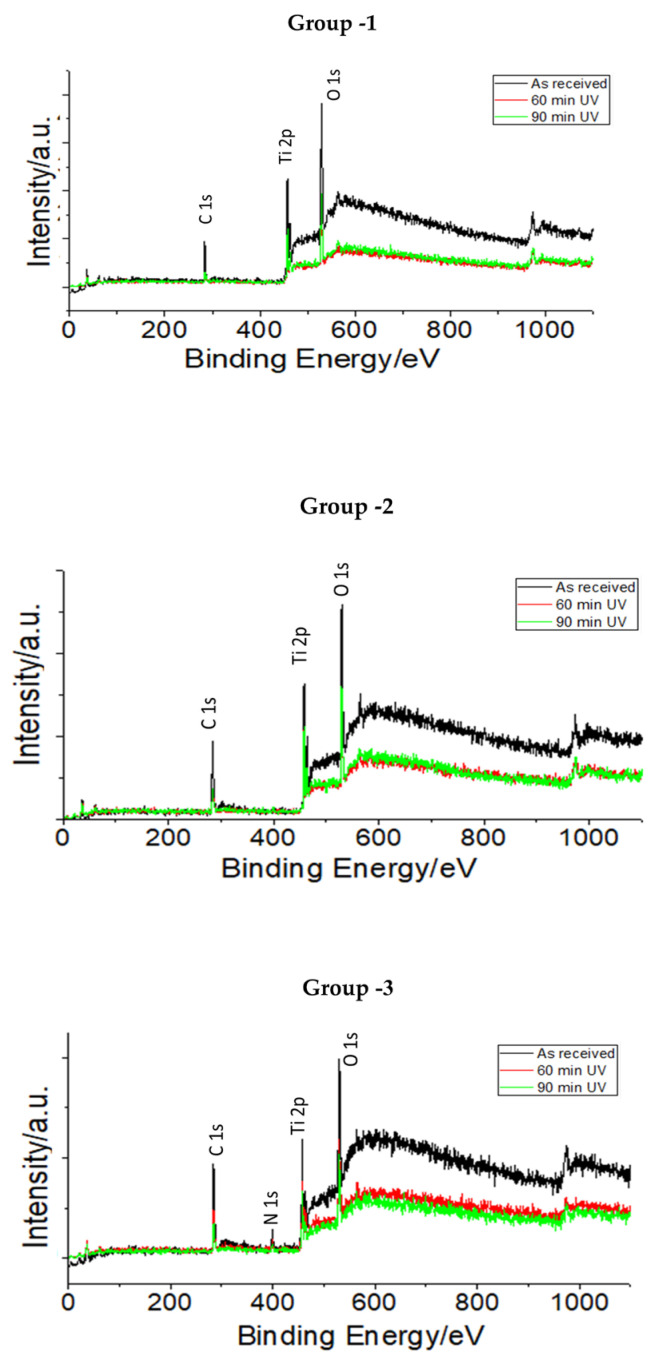
XPS survey spectra of various titanium surfaces before and after UV photofunctionalization.

The tests were conducted in triplicates for each assessment. Thus, with the sample size being small, the results were not statistically significant, although we can appreciate the alteration in the elements analyzed in the study group. In addition, the probing depth of XPS was 8–10 nm, and changes observed before and after UV irradiation demonstrated a change in the surface composition only. The changes in surface concentration of various elements before and after UV irradiation are summarized in Figure 1. The values shown in Table 1 indicate the signal density.

### 3.2. XPS Core-Level Spectra of C, O, and Ti in the Study Groups

**Figure 2:**
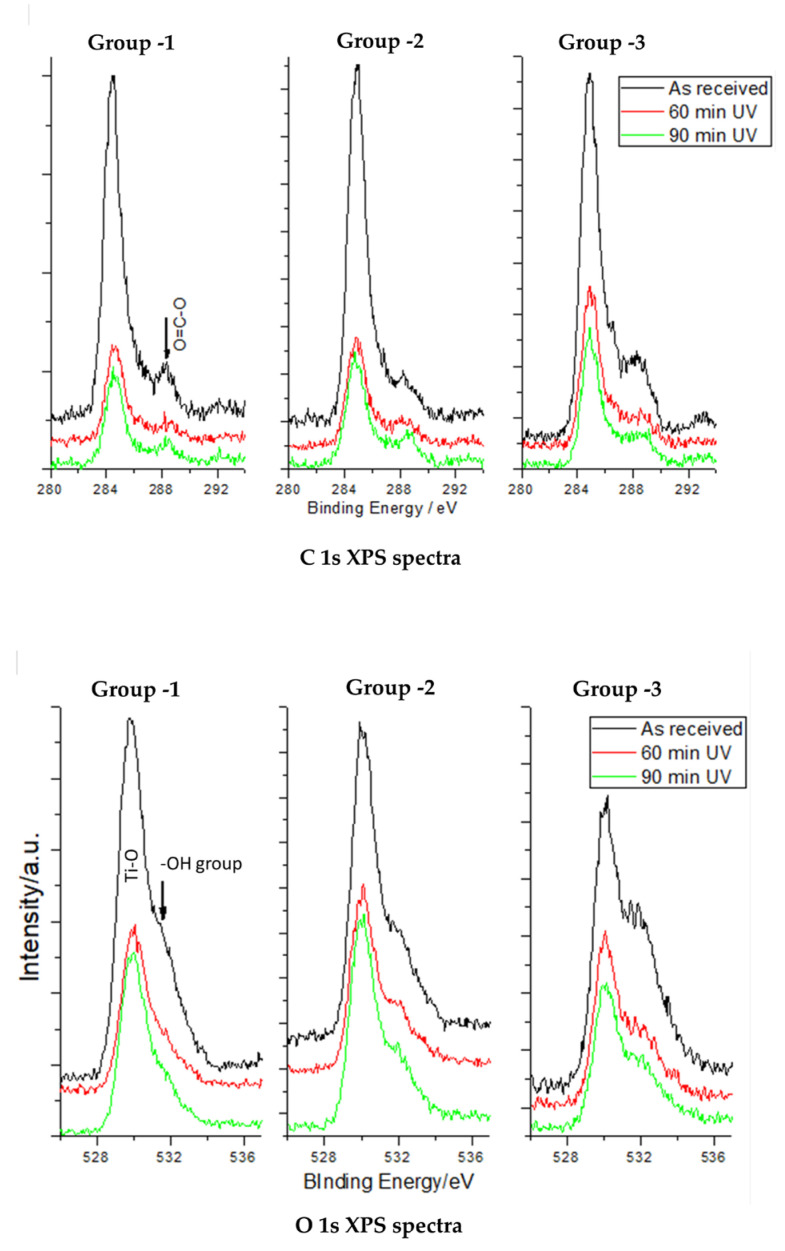

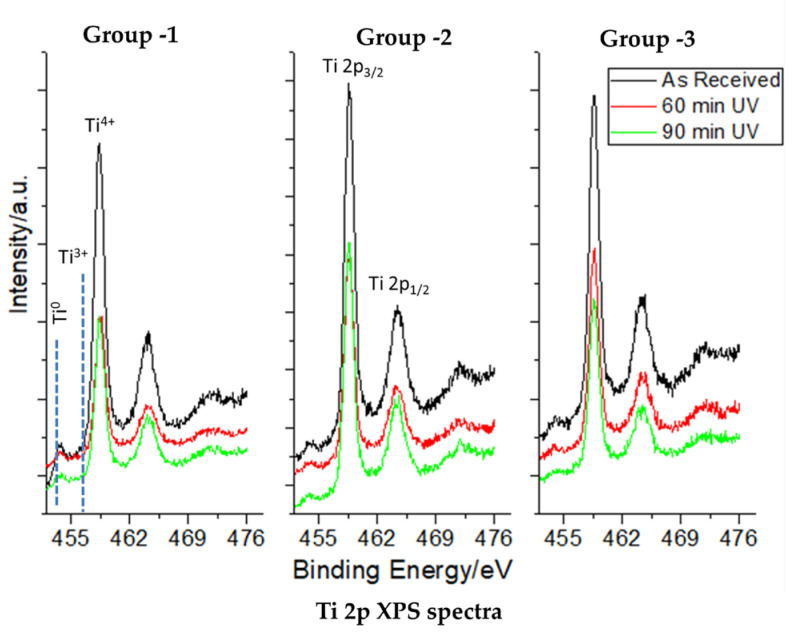
Change in surface concentrations of O 1s, C 1s and Ti 2p core level spectra of disc implants before and after UV photofunctionalization.

The chemical alteration of the titanium surface plays an important role in osseointegration. This is attributed to the conversion of the titanium (IV) to titanium (III) and formation of Ti-OH on the surface. High-resolution spectra of the Ti2p core level show two distinct peaks at 458.5 and 464.3 eV for Ti2p3/2 and Ti2p1/2, respectively, for the photofunctionalized group samples (Figure 2). A systematic deconvolution was carried out to evaluate the change in the percentage of various Ti species present on the surface of all the study group implant surfaces. The peak deconvolution confirms the presence of Ti (IV) species as a major peak component; however, Ti (III), Ti (II), and Ti (0) were also found as minor peak components. The ratio of various peaks differs in the different study groups. All three groups showed a decrease in the peak area of Ti (IV) species, which was followed by the corresponding enhancement of Ti (III), Ti (II), and Ti (0) peaks. The above observation confirms that the UV treatment of all the groups induced the reduction of Ti (IV) species. (Figure 2)

### 3.3. Morphology and Spreading Behavior of Osteoblasts Using Phase-Contrast Microscopy

**Figure 3:**
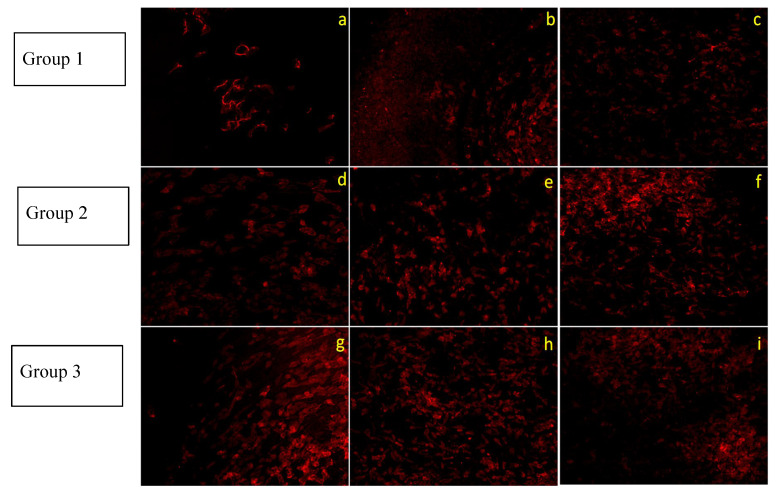
Confocal microscopic images of MG-63 osteoblasts cultured on UV photofunctionalized surfaces (Groups 1–3) for 24 h under high-glucose condition. Image (**a**–**c**) are control, 60 and 90 min UV-PF implants of Group 1. Images (**d**–**f**) are control, 60 and 90 min UV-PF implants of Group 2. Images (**g**–**i**) are control, 60 and 90 min UV-PF implants of Group 3, respectively (magnification 10×).

Osteoblastic morphology was evident after actin filament staining using a fluorescent dye, for all the implant surface groups, after photofunctionalization (Figure 3).

**Figure 4:**
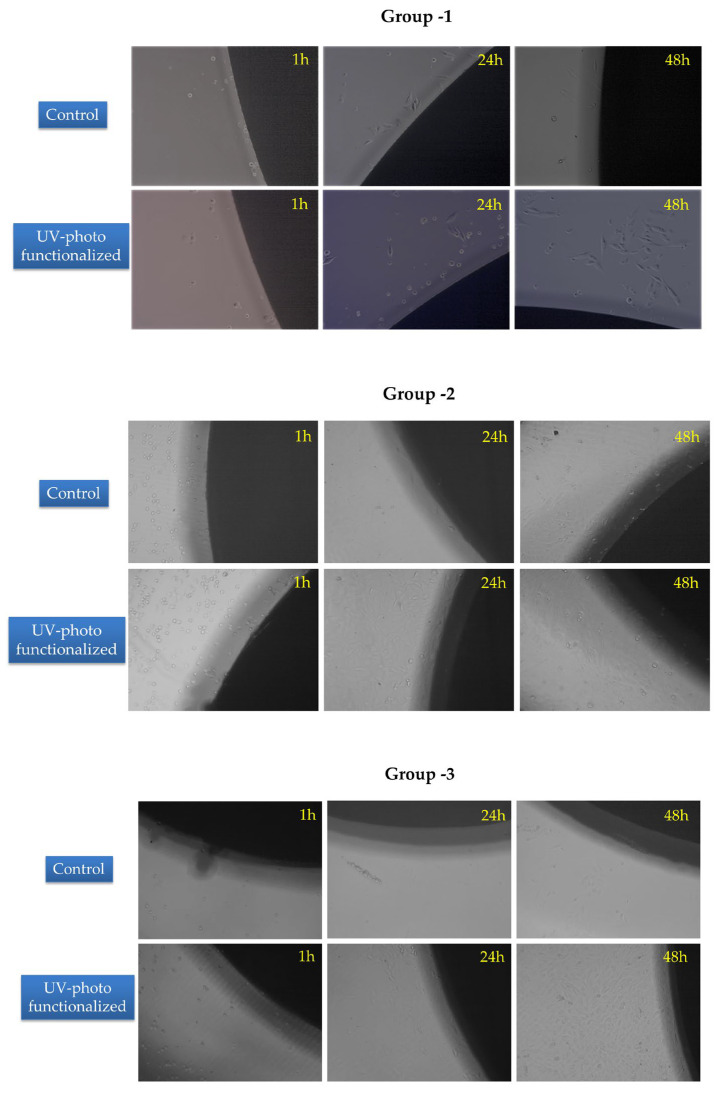
Phase contrast microscopic images of MG-63 osteoblasts grown in the presence of control and UV-PF surfaces (Group 1–3) in the presence of high-glucose concentration.

Cell morphology and growth were found to be the same in Group 1 implant surfaces in the presence of a photofunctionalized implant, indicating that the presence of high-glucose and UV-PF implant did not increase and improve cell attachment. In the case of Group 2 and 3 implant surfaces, it was observed that UV-PF improved initial cell attachment. After 24 h of incubation, cells attained their typical osteoblast morphology as compared to the untreated surface where cells were yet to attain their typical morphology. In addition, the number of cells attached to the photofunctionalized surfaces seemed to be more when compared to the control surface (Figure 4).

### 3.4. Cell Viability Assay for the Dental Implant Surfaces

Cell viability was assessed at 48 h for the control and the test groups (UV-treated implant surfaces). The test group at 48 h of incubation showed statistically higher cell viability than the control group. Only the 90 min UV-treated implants from Groups 1 (*p* < 0.05) and 3 (*p* < 0.05) showed significantly higher cell attachment compared to their untreated respective group. (Table 2)

### 3.5. Alizarin Red S Assay for the Dental Implant Surfaces

**Figure 5:**
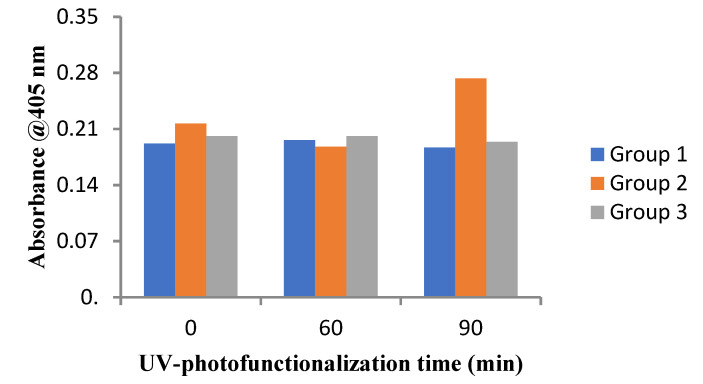
Assessment of mineralization using Alizarin Red assay on UV photofunctionalized titanium implant discs of various study groups in high-glucose medium.

Quantification of the ARS staining showed that the amount of ARS (calcium deposits) was more in the test group than in the control group after 21 days of incubation. There was no statistical difference in any group between control and 60 min UV application. Only in 90 min UV-treated Group 2 did implant surfaces show a statistically higher mineralization than the corresponding control samples. (Figure 5).

## 4. Discussion

The key findings of the present study revealed a significant effect of UV photofunctionalization on the reduction of carbon content in all the examined surfaces, greater cell attachment, and spreading of osteoblasts, along with more mineralization in a simulated diabetic microenvironment. Together, these findings support the potential of UV photofunctionalization treatment to benefit implant osseointegration in the compensated milieu of a diabetic state.

Photofunctionalization increases the hydrophilicity of surfaces broadly by two mechanisms: conversion of Ti4+ to Ti3+, which leads to the formation of titanium hydroxide groups, thereby generating oxygen vacancies that react with absorbed water [26]. The second mechanism is the elimination of the accumulated carbonyl moieties, especially hydrocarbons, from the surface [2,24,27]. Titanium surfaces are generally covered by carbon-containing molecules, the concentrations of which tend to increase during surface preparation and storage. The amount of surface carbon is known to increase to approximately 60% to 75% over some time as part of the aging of the implant surface. Varying amounts of contributions to the C1s peak are often found at other higher binding energies (286–290 eV), which is due to the presence of other bond types in the surface layer such as C-O and C-OH bonds [28,29]. Once the samples were irradiated with UV light, the relative UV treatment was found to reduce the carbon content and the contamination of the titanium surface by four times [30], and it has been observed that there is a direct correlation between surface carbon content and hydrophilicity of titanium surfaces [31].

In the present study, laser-etched implant surfaces (Group 1) showed the most reduction in carbon content upon UV treatment, i.e., 63% after 90 min and around 48% after 60 min. The air-abraded, acid-etched surfaces (Group 3) showed 19% reduction after 60 min and around 29% after 90 min of UV treatment. This is very close to observations reported earlier, showing a 20% reduction of carbon content after photofunctionalization on acid-etched titanium surfaces. Similarly, the titanium–zirconium alloy surface (Group 2) showed 54.5 and 38.5% reduction in carbon after 90 and 60 min of UV-PF, respectively. The XPS analysis conducted in the study showed significant reduction in the C1s peak with corresponding increases in the Ti2p and O1s peaks on all the examined surfaces after the UV treatment. There was also a decrease in Ti (IV) values on all surfaces. A formation of a thin TiO_2_ film was observed, which displayed more resistance to corrosion, and at a physiological pH value, it exhibited thermodynamical stability [32,33,34,35]. UV-treated titanium surfaces are also known to shift from biologically inert titanium to bioactive surfaces, which was supported by our cell culture experimental findings. The energy carried by UV rays breaks the contaminant molecules’ bonds from Ti atoms to O and/or N atoms, thereby increasing TiOH, which in turn is responsible for improved biocompatibility of the implant [30]. Our study groups showed a slight increase in oxygen peak after 90 min of UV photofunctionalization. There was a consistent increase in titanium peaks across all three surfaces and conversion into other Ti species such as Ti(3+) from Ti(4+).

The magnitude of photofunctionalization effect on different test surfaces was different in the present study. This was plausibly due to different biological characteristics of individual surface properties. Overall, the data supported that photofunctionalization at both time intervals (60 and 90 min) probably led to increased hydrophilicity of the treated surfaces, leading to better biological responses.

For effective osseointegration, attachment and proliferation of osteogenic cells is a primary requisite. Different surface topographies, including acid-etched, air-abraded, machined, and nano-featured surfaces were investigated in their studies [2,35,36]. Aligned with earlier reports, the number of osteoblasts attached to photofunctionalized surfaces was three- to five-fold higher than that attached to the untreated surfaces, especially for 3–24 h of incubation [2,9,24,27]. The phenomenon of increased biocompatibility and osteoblastic adherence to the implant surface has been attributed to a decrease in the amount of surface carbon molecules. Previously a negative correlation of surface carbon content with protein adsorption, cell attachment, cell spreading, and reduced calcium mineralization by osteoblasts was noted [37]. In the present study, a reduction in surface carbon content was accompanied by increased osteoblastic adherence.

Superior osteoblastic adherence and proliferation were observed on the SLA surface after photofunctionalization. This was in accordance with earlier reports, as osteocalcin was reported to have the highest secretion at mod SLA or hydrophilized SLA surfaces in a previous study, where osteogenic response of MG63 cells to different surface modifications was evaluated [3]. The effect of different implant surface treatments, including mechanical abrasion, sandblasting + acid etching, sandblasting, and acid-etching surfaces, on cell viability in vivo and cell lines demonstrated high cell viability and adherence of osteoblasts on sandblasted surfaces [38]. Similarly, better cell attachment and adherence were observed on surfaces combining acid etching and grit blasting [39].

Irrespective of surface morphology, a higher osteoblastic proliferation was observed after photofunctionalization despite the detrimental effect of the high-glucose medium. All Surfaces demonstrated significantly higher cell attachment at 48 h. Earlier evidence shows that UV-treated surfaces induce a 20–50% augmentation of the proliferation compared to control group [32,35].

The potential of the osteogenic cells to proliferate and differentiate determines the bone formation speed and extent. Effects of photofunctionalization were well-documented using both in vitro and in vivo models [40,41,42]. The photofunctionalization process increased the osteogenic response on acid-etched or sandblasted surfaces [4]. The other surfaces tested included Ti alloys (Ti6Al4V and Ti-Ag), SLA, machined surfaces, micro-arc oxidation surface, and Ti nanotubes. In such cases, photofunctionalization had augmented mineralization [10,26,32,43]. In a previous study, UV-induced cell attachment was observed on day 2 and the greatest mineralization was reached after 21 days [2]. In contrast, higher mineralization in the Titanium-Zirconia surfaces upon photofunctionalization were observed in the present study. Notably, all the treated implant surfaces demonstrated higher Alizarin Red activity as compared to untreated surfaces in the high-glucose medium as reported earlier [26,44].

The present study showed that photofunctionalization altered implant surface chemistry, making the surfaces more hydrophilic and conducive to increased osteoblastic adherence. Photofunctionalization also enhanced osteoblastic cell proliferation, especially after 48 h, and increased mineralization. All five test implant surfaces exhibited a reduction in carbon content, conversion of Ti4+ to Ti3+, increased osteoblastic adhesion and viability in the stressed tissue medium, and increased mineralization in a simulated diabetic environment. The combination of chemical treatment through UV photofunctionalization with manufacturer-designed nano- and micro-surface modifications can increase osteoblastic response despite a diabetogenic environment. These in vitro data provide a basis for pre-clinical and clinical studies using randomized controlled designs to investigate how photofunctionalization may benefit dental implant outcomes in diabetes.

One limitation of the study was that the sample size was small for each group. Another limitation was that there are other implants with different surface features that may react differently to photofunctionalization. Therefore, other implant surfaces should also be evaluated to better understand their success in a high-glucose environment.

## 5. Conclusions

Photofunctionalization alters the surface chemistry of dental implant surfaces, enhancing hydrophilicity, and leading to increased osteoblastic adherence, cell proliferation, and mineralization in a high-glucose environment. Based on the results of this study, it can be suggested that UV photofunctionalization could be adopted to enhance dental implant osteointegration, especially in diabetic patients who are particularly at high risk for implant failures.

## 6. Clinical Relevance

Photofunctionalization favored osteoblast attachment and mineralization on the implant surface, which in turn is responsible for the increased initial osteointegration. In some systemic conditions, such as in diabetes mellitus type II patients, photofunctionalization of implant surfaces prior to their placement could increase clinical success and longevity of the implant. More clinical research in this direction could further validate our findings.

## Figures and Tables

**Table 1:** XPS surface analysis of all groups with different UV application time.

	Control (Mean ± SD)	60 min (Mean ± SD)	90 min (Mean ± SD)
C 1s			
Group 1	20.63 ± 0.058	10.10 ± 0.100	7.50 ± 0.033
Group 2	20.77 ± 0.058	12.76 ± 0.100	9.45 ± 0.033
Group 3	22.67 ± 0.580	18.50 ± 0.580	16.20 ± 0.010
O 1s			
Group 1	40.60 ± 0.100	43.87 ± 0.58	43.80 ± 0.100
Group 2	40.33 ± 0.577	42.70 ± 0.100	44.60 ± 0.100
Group 3	41.53 ± 0.153	41.76 ± 0.058	44.10 ± 0.100
Ti 2p			
Group 1	37.55 ± 0.306	43.30 ± 0.100	46.07 ± 0.058
Group 2	39.20 ± 0.100	41.80 ± 0.100	42.33 ± 0.058
Group 3	31.40 ± 0.100	34.65 ± 0.026	36.67 ± 0.577

**Table 2:** MTT assay for cell viability.

	Baseline Mean (%) ± SD	90 min Mean (%) ± SD
Group 1	100 ± 3 ^a^	125 ± 3.75 ^b^
Group 2	100 ± 3 ^a^	110.04 ± 3.30 ^a^
Group 3	100 ± 3 ^a^	125 ± 3.75 ^b^

The letters “a” and “b” indicate significant differences both between the rows and the columns.

## Data Availability

Data is contained within the article. The data presented in this study are available in [In Vitro Evaluation of Photofunctionalized Implant Surfaces in a High-Glucose Microenvironment Simulating Diabetics].

## References

[1] Rupp F., Liang L., Geis-Gerstorfer J., Scheideler L., Hüttig F. (2017). Surface characteristics of dental implants: A review. Dent. Mater.

[2] Aita H., Hori N., Takeuchi M., Suzuki T., Yamada M., Anpo M., Ogawa T. (2009). The effect of ultraviolet functionalization of titanium on integration with bone. Biomaterials.

[3] Gittens R.A., Scheideler L., Rupp F., Hyzy S.L., Geis-Gerstorfer J., Schwartz Z., Boyan B.D. (2014). A review on the wettability of dental implant surfaces II: Biological and clinical aspects. Acta Biomater..

[4] Smeets R., Stadlinger B., Schwarz F., Beck-broichsitter B., Jung O., Precht C., Kloss F., Gröbe A., Heiland M., Ebker T. (2016). Impact of Dental Implant Surface Modifications on Osseointegration. Biomed Res. Int..

[5] Sugita Y., Honda Y., Kato D.D.S.I. (2014). Role of Photofunctionalization in Mitigating Impaired Osseointegration Associated with Type 2 Diabetes in Rats. Int. J. Oral Maxillofac. Implants.

[6] Wang C.K., Lin J.H., Ju C.P., Ong H.C., Chang R.P. (1997). Structural characterization of pulsed laser-deposited hydroxyapatite film on titanium substrate. Biomaterials.

[7] Carossa M., Cavagnetto D., Mancini F., Mosca Balma A., Mussano F. (2022). Plasma of Argon Treatment of the Implant Surface, Systematic Review of In Vitro Studies. Biomolecules.

[8] Dini C., Nagay B.E., Magno M.B., Maia L.C., Barão V.A.R. (2020). Photofunctionalization as a suitable approach to improve the osseointegration of implants in animal models—A systematic review and meta-analysis. Clin. Oral Implants Res..

[9] Funato A., Yamada D.D.S.M., Ogawa T. (2013). Success Rate, Healing Time, and Implant Stability of Photofunctionalized Dental Implants. Int. J. Oral Maxillofac. Implants.

[10] Funato A., Ogawa T. (2013). Photofunctionalized Dental Implants: A Case Series in Compromised Bone. Int. J. Oral Maxillofac. Implants.

[11] Hirakawa Y., Jimbo R., Shibata Y., Watanabe I., Wennerberg A., Sawase T. (2013). Accelerated bone formation on photo-induced hydrophilic titanium implants: An experimental study in the dog mandible. Clin. Oral Implants Res..

[12] Ikeda T., Okubo T., Saruta J., Hirota M., Kitajima H., Yaagisawa N., Ogawa T. (2021). Osteoblast attachment compromised by high and low temperature of titanium and its restoration by UV photofunctionalization. Materials.

[13] Okonkwo U., DiPietro L. (2017). Diabetes and Wound Angiogenesis. Int. J. Mol. Sci..

[14] Kanazawa I., Yamaguchi T., Yamamoto M., Yamauchi M., Kurioka S., Yano S., Sugimoto T. (2009). Serum Osteocalcin Level Is Associated with Glucose Metabolism and Atherosclerosis Parameters in Type 2 Diabetes Mellitus. J. Clin. Endocrinol. Metab..

[15] Krakauer J.C., Mckenna M.J., Buderer N.F., Rao D.S., Whitehouse F.W., Parfitt A.M. (1995). Bone Loss and Bone Turnover in Diabetes. Diabetes.

[16] Pedrazzoni M., Ciotti G., Pioli G., Girasole G., Davoli L., Palummeri E., Passeri M. (1989). Osteocalcin levels in diabetic subjects. Calcif. Tissue Int..

[17] Shu A., Yin M.T., Stein E., Cremers S., Dworakowski E., Ives R., Rubin M.R. (2012). Bone structure and turnover in type 2 diabetes mellitus. Osteoporos. Int..

[18] Plotkin L.I., Bellido T. (2016). Osteocytic signalling pathways as therapeutic targets for bone fragility. Nat. Rev. Endocrinol..

[19] Javed F., Romanos G.E. (2019). Chronic hyperglycemia as a risk factor in implant therapy. Periodontology 2000.

[20] Moraschini V., Barboza E.S.P., Peixoto G.A. (2016). The impact of diabetes on dental implant failure: A systematic review and meta-analysis. Int. J. Oral Maxillofac. Surg..

[21] Jiang X., Zhu Y., Liu Z., Tian Z., Zhu S. (2021). Association between diabetes and dental implant complications: A systematic review and meta-analysis. Acta Odontol. Scand..

[22] Al Ansari Y., Shahwan H., Chrcanovic B.R. (2022). Diabetes Mellitus and Dental Implants: A Systematic Review and Meta-Analysis. Materials.

[23] Pacicca D.M., Brown T., Watkins D., Kover K., Yan Y., Prideaux M., Bonewald L. (2019). Elevated glucose acts directly on osteocytes to increase sclerostin expression in diabetes. Sci. Rep..

[24] Ogawa T. (2014). Ultraviolet Photofunctionalization of Titanium Implants. Int. J. Oral Maxillofac. Implants.

[25] Chauhan M., Reddy K.P., Gopinath C.S., Deka S. (2017). Copper cobalt sulphide nanosheets realizing a promising electrocatalytic oxygen evolution reaction. ACS Catal..

[26] Gao Y., Liu Y., Zhou L., Guo Z., Rong M., Liu X., Lai C., Ding X. (2013). The Effects of Different Wavelength UV Photofunctionalization on Micro-Arc Oxidized Titanium. PLoS ONE.

[27] Att W., Hori N., Takeuchi M., Ouyang J., Yang Y., Anpo M., Ogawa T. (2009). Time-dependent degradation of titanium osteoconductivity: An implication of biological aging of implant materials. Biomaterials.

[28] Kang B.S., Sul Y.T., Oh S.J., Lee H.J., Albrektsson T. (2009). XPS, AES and SEM analysis of recent dental implants. Acta Biomater..

[29] Sawase T., Hai K., Yoshida K., Baba K., Hatada R., Atsuta M. (1998). Spectroscopic studies of three osseointegrated implants. J. Dent..

[30] Roy M., Pompella A., Kubacki J., Szade J., Roy R.A., Hedzelek W. (2016). Photofunctionalization of Titanium: An Alternative Explanation of Its Chemical-Physical Mechanism. PLoS ONE.

[31] Takeuchi M., Sakamoto K., Martra G., Coluccia S., Anpo M. (2005). Mechanism of Photoinduced Superhydrophilicity on the TiO_2_ Photocatalyst Surface. J. Phys. Chem. B.

[32] Hori N., Ueno T., Minamikawa H., Iwasa F., Yoshino F., Kimoto K., Lee M.C.-I., Ogawa T. (2010). Electrostatic control of protein adsorption on UV-photofunctionalized titanium. Acta Biomater..

[33] Ogawa T., Anpo M., Kamat P. (2010). Photofunctionalization of TiO2 for Optimal Bone-titanium Integration: A Novel Phenomenon of Super Osseointegration. Environmentally Benign Photocatalysts. Nanostructure Science and Technology.

[34] Ogawa T., Iwasa F., Tsukimura N., Att W., Kodali-Kanuru R., Kubo K., Hasnain H. (2011). TiO2 micro-nano-hybrid surface to alleviate biological aging of UV-photofunctionalized titanium. Int. J. Nanomed..

[35] Tsukimura N., Yamada M., Iwasa F., Minamikawa H., Att W., Ueno T., Saruwatari L., Aita H., Chiou W.-A., Ogawa T. (2011). Synergistic effects of UV photofunctionalization and micro-nano hybrid topography on the biological properties of titanium. Biomaterials.

[36] Iwasa F., Hori N., Ueno T., Minamikawa H., Yamada M., Ogawa T. (2010). Enhancement of osteoblast adhesion to UV-photofunctionalized titanium via an electrostatic mechanism. Biomaterials.

[37] Hayashi R., Ueno T., Migita S., Tsutsumi Y., Doi H., Ogawa T., Hanawa T., Wakabayashi N. (2014). Hydrocarbon Deposition Attenuates Osteoblast Activity on Titanium. J. Dent. Res..

[38] Velasco-Ortega E., Alfonso-Rodríguez C.A., Monsalve-Guil L., España-López A., Jiménez-Guerra A., Garzón I., Alaminosn M., Gil F.J. (2016). Relevant aspects in the surface properties in titanium dental implants for the cellular viability. Mater. Sci. Eng. C Mater. Biol. Appl..

[39] Sammons R.L., Lumbikanonda N., Gross M., Cantzler P. (2005). Comparison of osteoblast spreading on microstructured dental implant surfaces and cell behaviour in an explant model of osseointegration. Clin. Oral Implants Res..

[40] Choi B., Lee Y.C., Oh K.C., Lee J.H. (2021). Effects of photofunctionalization on early osseointegration of titanium dental implants in the maxillary posterior region: A randomized double-blinded clinical trial. Int. J. Implant Dent..

[41] Chang L.C. (2022). Clinical Applications of Photofunctionalization on Dental Implant Surfaces: A Narrative Review. J. Clin. Med..

[42] Roy M., Corti A., Dorocka-Bobkowska B., Pompella A. (2022). Positive Effects of UV-Photofunctionalization of Titanium Oxide Surfaces on the Survival and Differentiation of Osteogenic Precursor Cells—An In Vitro Study. J. Funct. Biomater..

[43] Minamikawa H., Ikeda T., Att W., Hagiwara Y., Hirota M., Tabuchi M., Aita H., Park W., Ogawa T. (2014). Photofunctionalization increases the bioactivity and osteoconductivity of the titanium alloy Ti6Al4V. J. Biomed. Mater. Res. A.

[44] Suzuki T., Hori N., Att W., Kubo K., Iwasa F., Ueno T., Maeda H., Ogawa T. (2009). Ultraviolet Treatment Overcomes Time-Related Degrading Bioactivity of Titanium. Tissue Eng. Part A.

